# Rosmarinic acid suppresses tau phosphorylation and cognitive decline by downregulating the JNK signaling pathway

**DOI:** 10.1038/s41538-021-00084-5

**Published:** 2021-01-29

**Authors:** So Yamamoto, Tomoko Kayama, Moeko Noguchi-Shinohara, Tsuyoshi Hamaguchi, Masahito Yamada, Keiko Abe, Shoko Kobayashi

**Affiliations:** 1 Research Center for Food Safety, Graduate School of Agricultural and Life Sciences, The University of Tokyo, Bunkyo-ku, Tokyo Japan; 2 Department of Neurology and Neurobiology of Aging, Kanazawa University Graduate School of Medical Sciences, Takara-machi, Kanazawa Japan; 3 Department of Applied Biological Chemistry, Graduate School of Agricultural and Life Sciences, The University of Tokyo, Yayoi, Bunkyo-ku, Tokyo Japan; 4 Group of Food Functionality Assessment, Kanagawa Institute of Industrial Science and Technology, Life Science Environment Research Center, Tonomachi, Kawasaki, Kanagawa Japan

**Keywords:** Quality of life, Diseases

## Abstract

Rosmarinic acid (RA), a polyphenol found in Lamiaceae herbs, is a candidate of preventive ingredients against Alzheimer’s disease (AD) as it potently suppresses the aggregation of amyloid β (Aβ); however, the effect of RA on tau phosphorylation and cognitive dysfunction remains unclear. The present study revealed that RA intake inhibited the pathological hallmarks of AD, including Aβ and phosphorylated tau accumulation, and improved cognitive function in the 3 × Tg-AD mouse model. Additionally, RA intake suppressed hippocampal inflammation and led to the downregulation of the JNK signaling pathway that induces tau phosphorylation. Feeding with RA exerted an anti-inflammatory effect not only in the central nervous system but also in the periphery. Downregulation of the JNK signaling pathway in hippocampus may be a potential mechanism underlying the inhibition of progression of pathology and cognitive deficit by RA feeding.

## Introduction

Alzheimer’s disease (AD) is clinically characterized as progressive dementia with the pathological hallmarks of senile plaques and neurofibrillary tangles comprising amyloid-β peptide (Aβ) and hyperphosphorylated tau (p-tau), respectively. The currently available drugs temporarily relieve symptoms without providing cure for AD. Therefore, food components that can contribute to the prevention of AD are attracting attention.

**Fig. 1:**
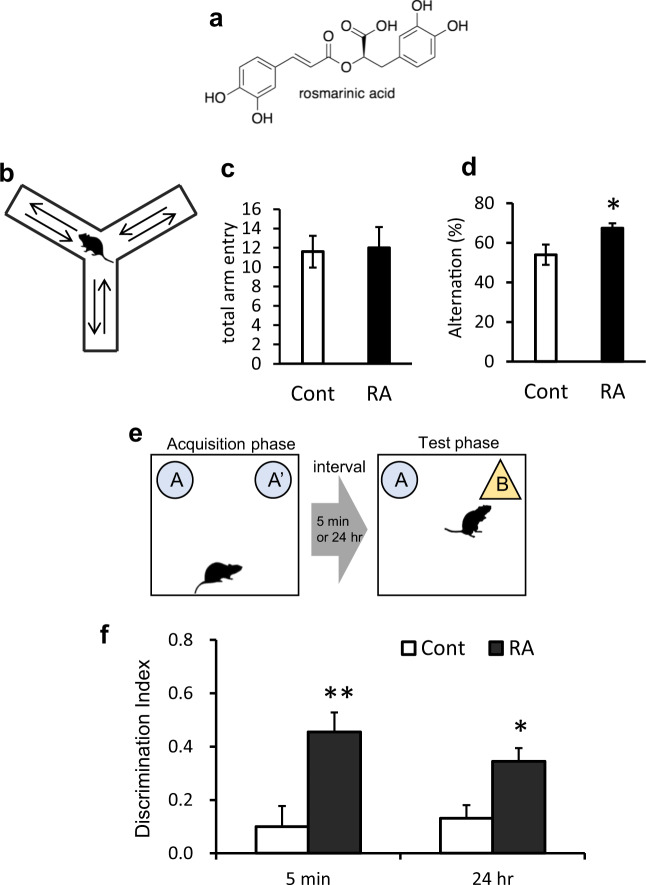
RA improve cognitive function in 3 × Tg-AD mice. **a** The structure of rosmarinic acid (RA). **b**–**d** Spatial memory is improved by feeding with an RA-supplemented diet. **b** Y-maze test scheme. **c** The number of arm entries are similar in both groups. **d** The spatial memory is lower in the control (Cont) group. **e**–**f** Memory for objects is improved in mice fed an RA-supplemented diet. **e** Acquisition and test scheme for novel object recognition test (NORT). **f** NORT suggests RA improved cognitive function for object recognition. Data are shown as means ± standard error. Cont, *n* = 8; RA, *n* = 9, *p < 0.05, **p < 0.01.

Several epidemiological studies indicate that a polyphenol-rich diet reduces the risk of AD^1,2^. In particular, rosmarinic acid (RA) is a promising anti-AD agent because it strongly inhibits Aβ aggregation and alleviates synaptic toxicity in vitro^3^. RA binds to the β-sheet structure of Aβ and directly inhibits its aggregation in vitro^3^. RA is a phenylpropanoid polyphenol found in members of the Lamiaceae herb family such as rosemary and lemon balm (Fig. 1a).

We previously reported that RA as a diet supplement inhibited Aβ oligomerization in Tg2576 mice, a model of AD^4^. We also reported a mechanism of the suppression of Aβ aggregation in RA-fed mice^5^. However, it remained unclear whether the effect of RA on tau phosphorylation and cognitive dysfunction. Phosphorylated tau has strong neurotoxicity and induces neuronal death^6^. The major neuropathological lesions of AD are extracellular deposits of Aβ peptides leading to the formation of senile/neuritic plaques and intercellular NFTs, which are paired helical filaments of hyperphosphorylated tau protein^7^. The effects of RA have been demonstrated in several studies using drug-induced cognitive deficits and intraventricular Aβ injection in animals^8,9^. Although RA was demonstrated to suppress chronic restraint stress-induced tau aggregation^10^, it remains unclear the molecular mechanism and whether RA intake also impacts tau pathology, which highly correlates with cognitive status in AD^11^. Most of the structure-based mechanisms by which RA inhibits the accumulation of Aβ or tau in vitro^12,13^ have been examined at higher concentrations of RA than that is expected to be transported to the brain. Thus, in vivo RA may function via a different mechanism that has been described in vitro.

The main purpose of the present study was to extensively characterize the mechanism underlying the preventive effect of RA in AD. We used 3 × Tg-AD mice, which allowed the examination of Aβ and p-Tau overexpression in the same strain^14^. Our evaluations included pathology and memory assessment of 3 × Tg-AD mice fed an RA-containing diet before the onset of AD pathology. First, we investigated the effect of RA on cognitive function in 3 × Tg-AD mice using multiple noninvasive behavioral tests for minimize the influence on subsequent transcriptomics analyses. We also visualized early pathological changes in the brains of 3 × Tg-AD mice by immunohistochemistry. In addition, we conducted transcriptome analysis in the 3 × Tg-AD mouse hippocampus, a key brain structure for learning and memory, for a comprehensive examination of the effects of RA intake.

## Results

### Effects of RA on body weight and plasma biochemical parameters

There was no between-group difference in food intake or body weight (Supplementary Fig. S1a, b). The plasma samples collected from all animals in both groups (*n* = 8 per group), after the exclusion of one hemolyzed sample in the RA group, were analyzed to determine potential changes in 12 biochemical parameters. The creatine kinase levels tended to be higher in the RA group while there were no significant differences in the levels of other plasma proteins between the two groups (Supplementary Table S2).

### RA improves the spatial memory of AD model mice

The behavioral Y-maze test to evaluate hippocampal spatial working memory revealed that the total number of arm entries did not change between the RA and control groups, indicating that there was no change in locomotor activity between the groups (Fig. 1c). However, alternation ratio was higher in the RA group than in the control group (Fig. 1d). These results suggested that the RA group exhibited improved behavioral performance related to spatial working memory compared to the control group.

### RA improves nonspatial cognitive deficit in the novel object recognition test

We next used the novel object recognition test to evaluate the spatially independent memory for objects. In the acquisition phase, no significant difference was observed in the exploratory behavior time for two objects in neither group (data not shown). The RA group showed a higher DI than the control group both at 5 min and 24 h in the test phase (Fig. 1f). These results suggested that the RA group improved behavioral performance related to memory for objects compared to the control group.

### RA delays the progression of AD pathology in 3 × Tg-AD mice

**Fig. 2:**
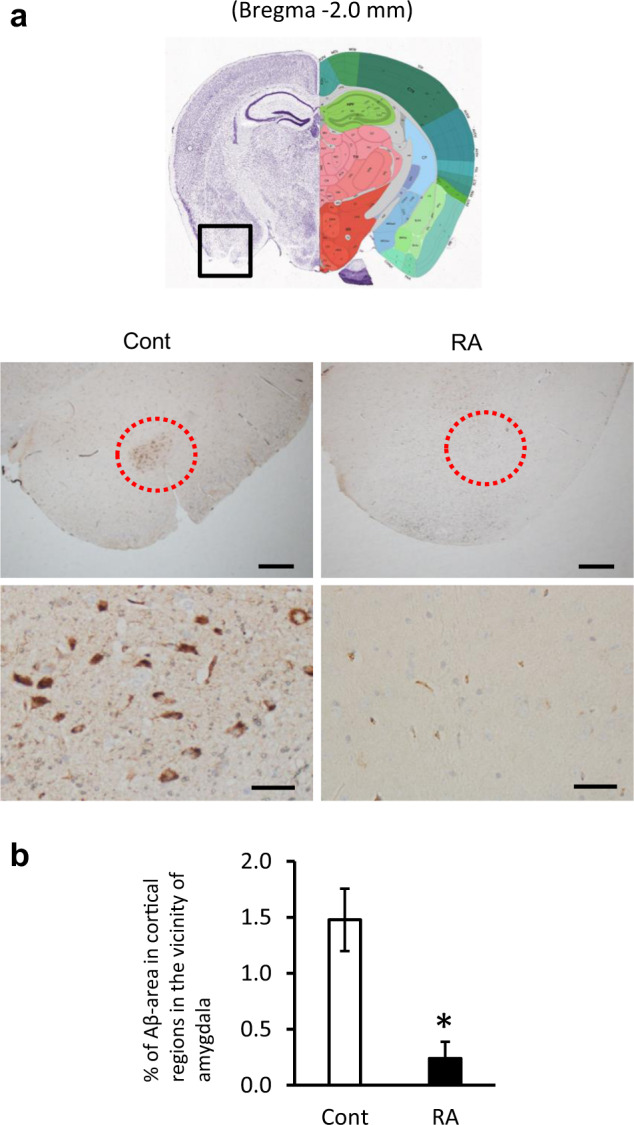
Suppression of Aβ accumulation in Alzheimer’s disease pathology by RA intake. Paraffin sections were stained with anti-Aβ (4G8) and peroxidase-based labeling (brown) and counterstained with hematoxylin to visualize nuclei (violet). **a** In the RA group, Aβ accumulation was reduced in cortical regions in the vicinity of the amygdala. Images in bottom are the magnified view of the red circle in the upper figure. The scale bars in upper and lower showed 500 and 50 μm in distance, respectively. **b** Aβ plaque area per 1000 μm^2^ of cortical regions in the vicinity of the amygdala. Data are shown as means ± standard error. Cont, *n* = 3; RA, *n* = 3; *p < 0.05.

**Fig. 3:**
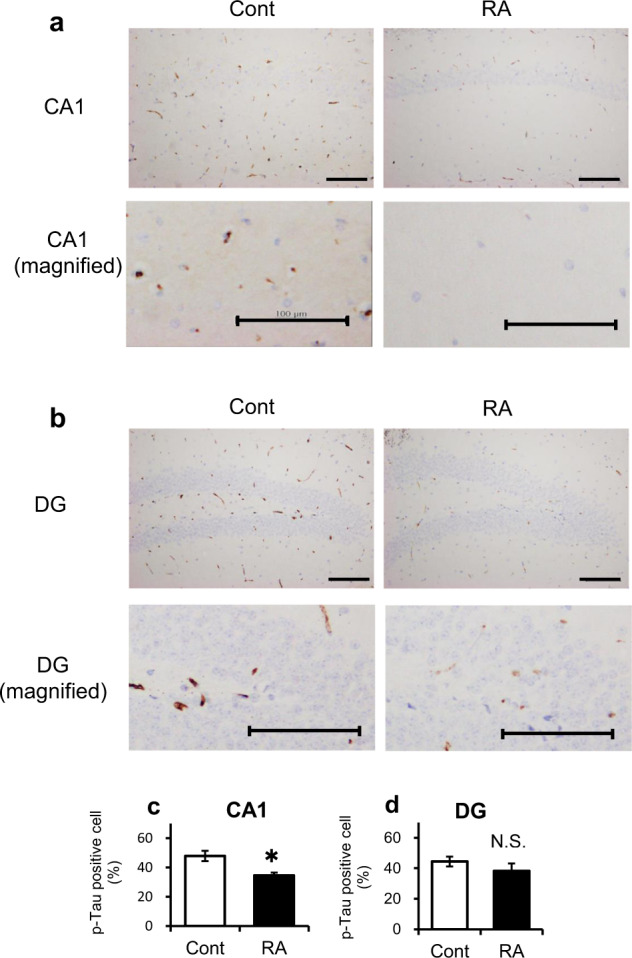
Suppression of p-tau in Alzheimer’s disease pathology by RA intake. Paraffin sections were stained with anti-p-tau (AT8) and peroxidase-based labeling (brown) and counterstained with hematoxylin to visualize nuclei (violet). Immunohistochemistry for p-tau in the CA1 (**a**) and DG (**b**) regions of the hippocampus. The scale bar showed 100 μm in distance. **c**–**d** Percentage of p-tau-positive cells in CA1 (**c**) and DG (**d**). Bregma, −1.8 to −2.2 mm; slice thickness, 5 μm Data are shown as means ± standard error. Cont, *n* = 3; RA, *n* = 3, *p < 0.05.

We performed immunohistochemistry to evaluate the expression of Aβ and p-tau, key molecules of AD progression. The accumulation of Aβ, which was observed in the control group’s cortical regions in the vicinity of the amygdala, was suppressed in the RA group (Fig. 2a, b); significant Aβ accumulation was not observed in any other brain region. In the control group, p-tau accumulation was observed surrounding the hippocampus. According to the proportion of p-tau-positive cells to the total number of cells in the hippocampus vs. the total number of cells, there was a significant decrease in the CA1 region of the RA group relative to the control group (Fig. 3a, c). In the dentate gyrus, the proportion of p-tau-positive cells did not significantly change between the two groups (Fig. 3b, d). Suppression of p-tau in the CA1 region responsible for hippocampal integrity might contribute to improvement in cognitive function.

### Feeding with RA changes the transcriptome of hippocampus

**Fig. 4:**
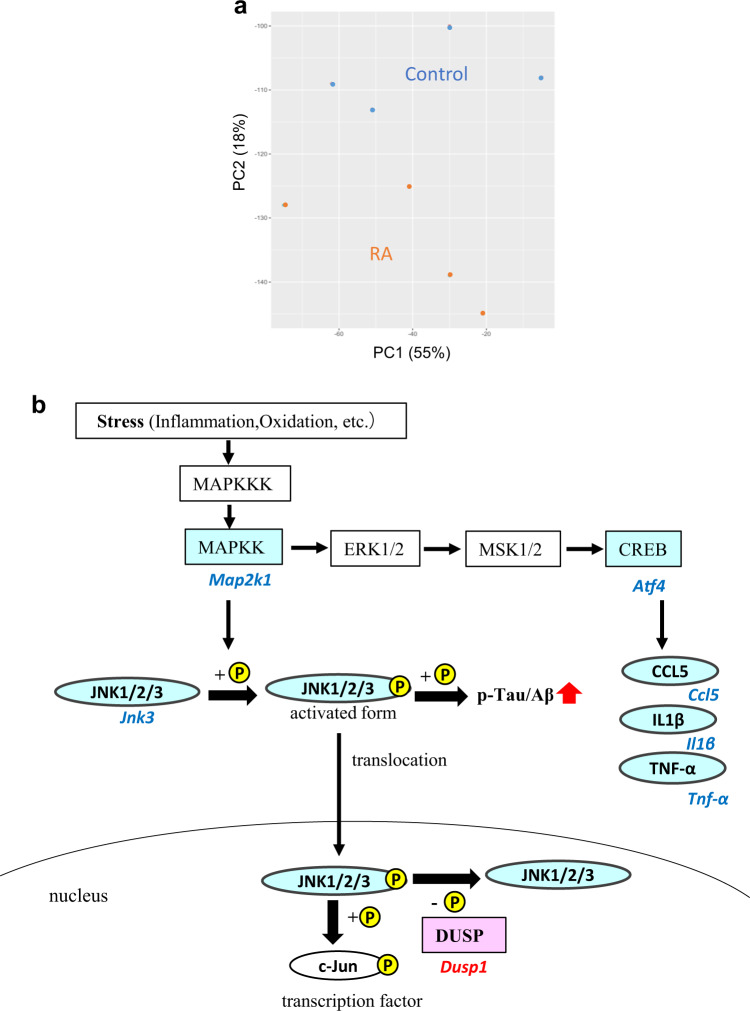
Microarray analysis of changes in hippocampal gene expression. **a** Principal component analysis (PCA) shows that two distinct clusters in hippocampal gene expression. **b** The annotated differentially expressed genes (DEGs) of c-Jun N-terminal kinase (JNK) signaling, which related to inflammation and tau phosphorylation. The DEGs annotated with KEGG pathway of “TNF signaling pathway” are shown in an integrated MAPK/JNK pathway. Italicized and nonitalicized letters indicate genes and proteins, respectively. Red and blue letters indicate genes that are up- and downregulated, respectively.

We next performed DNA microarray analysis of hippocampus because the number of p-tau-positive cells was decreased by RA feeding. The RA group showed a distinctly different gene expression profile to that of the control group (Fig. 4a). A total of 549 DEGs were identified (false discovery rate, 0.05) and annotated with GO terms to categorize their function (Table 1). The DEGs were annotated with GO terms related to nervous system development (e.g., nervous system development and substantia nigra development), memory (e.g., long-term synaptic potentiation), and neurotransmission (e.g., gamma-aminobutyric acid signaling pathway). These data suggested that nervous system development, memory, and neurotransmission changed in the RA group.

**Table 1:** Gene ontology terms enriched in the rosmarinic acid group.

Gene ontology	Annotated genes	Fold enrichment	*p-*value
GO:0007399 nervous system development	30	3.1	<0.0001
GO:0007568 aging	18	4.0	<0.0001
GO:0021762 substantia nigra development	8	8.1	<0.0001
GO:0060291 long-term synaptic potentiation	8	6.3	0.00025
GO:0007214 gamma-aminobutyric acid signaling pathway	6	9.3	0.00039
GO:0034341 response to interferon-gamma	6	8.9	0.00047
GO:0032922 circadian regulation of gene expression	8	5.1	0.00096
GO:0032355 response to estradiol	10	3.8	0.0012
GO:0032092 positive regulation of protein binding	8	4.5	0.0018
GO:0007623 circadian rhythm	10	3.6	0.0019
GO:0001822 kidney development	11	3.3	0.0020
GO:0032868 response to insulin	8	4.4	0.0023
GO:0042060 wound healing	9	3.7	0.0030
GO:0042572 retinol metabolic process	5	8.0	0.0031
GO:0006641 triglyceride metabolic process	6	5.9	0.0031
GO:0035455 response to interferon-alpha	4	12.9	0.0032
GO:0051384 response to glucocorticoid	8	4.1	0.0032
GO:0009416 response to light stimulus	5	7.7	0.0036
GO:0009749 response to glucose	8	4.0	0.0040
GO:1902476 chloride transmembrane transport	6	5.5	0.0044
GO:0007050 cell cycle arrest	8	3.8	0.0049

Enrichment analysis of 549 differentially expressed genes, with a false discovery rate of 0.05, has identified enrichment for 21gene ontologies (Biological Process). Significance criteria were a *p* value of <0.005 with the modified Fisher’s exact test and a fold enrichment score of >3.0. The terms are listed according to their *p* values.

To identify significantly enriched pathways, we conducted Kyoto Encyclopedia of Genes and Genomes (KEGG) pathway analysis in DAVID (Table 2). We focused on inflammation-related pathways such as the transforming growth factor-beta signaling pathway and the tumor necrosis factor (TNF) signaling pathway because of the chronic inflammation had reported in the brains of patients with AD^15^. Our investigation of the DEGs in the TNF signaling pathway revealed that mitogen-activated protein kinase (*Map2k1*), *Jnk3*, activating transcription factor (*Atf4*), and chemokine (C–C motif) ligand 5 (*Ccl5*) were downregulated. These data suggested that the MAPK/JNK signaling pathway annotated in “TNF signaling pathway” was downregulated in the RA group compared with the control group. JNK3 is one of the tau phosphorylation kinases^16^. We integrated the TNF signaling pathway and DEGs related to JNK3-mediated tau phosphorylation into a single pathway (Fig. 4b). Dual-specificity phosphatase (*Dusp1*), a phosphatase that invalidates the active JNK, was upregulated in the RA group. These results suggested that tau phosphorylation and Aβ aggregation were decreased via the inhibition of the MAPK signaling pathway, especially via a decrease in JNK3 activity.

**Table 2:** KEGG pathways enriched in the rosmarinic acid group.

KEGG pathway	Annotated genes	Fold enrichment	*p-*value
mmu04022: cGMP-PKG signaling pathway	19	3.7	<0.0001
mmu04723: Retrograde endocannabinoid signaling	13	4.2	<0.0001
mmu05210: Colorectal cancer	9	4.7	0.00056
mmu04915: Estrogen signaling pathway	11	3.8	0.00062
mmu04724: Glutamatergic synapse	11	3.2	0.00212
mmu01212: Fatty acid metabolism	7	4.6	0.00376
mmu04727: GABAergic synapse	9	3.5	0.00413
mmu05213: Endometrial cancer	7	4.5	0.00415
mmu04270: Vascular smooth muscle contraction	11	2.9	0.00437
mmu04114: Oocyte meiosis	10	3.1	0.00525
mmu05032: Morphine addiction	9	3.2	0.00619
mmu05033: Nicotine addiction	6	5.0	0.00620
mmu04728: Dopaminergic synapse	11	2.8	0.00637
mmu04978: Mineral absorption	6	4.9	0.00690
mmu04730: Long-term depression	7	3.9	0.00908
mmu04110: Cell cycle	10	2.7	0.01124
mmu04350: TGF-beta signaling pathway	8	3.2	0.01281
mmu04720: Long-term potentiation	7	3.6	0.01316
mmu05215: Prostate cancer	8	3.1	0.01530
mmu04668: TNF signaling pathway	9	2.8	0.01545
mmu04918: Thyroid hormone synthesis	7	3.4	0.01723
mmu04924: Renin secretion	7	3.3	0.01837
mmu04971: Gastric acid secretion	7	3.3	0.01956

Enrichment analysis of 549 differentially expressed genes, with a false discovery rate of 0.05, has identified enrichment for 23 KEGG pathways. Significance criteria were a *p* value of < 0.02 with the modified Fisher’s exact test and a fold enrichment score of >2.7. The terms are listed according to their *p* values.

### Feeding with RA modulates JNK expression in the brain

**Fig. 5:**
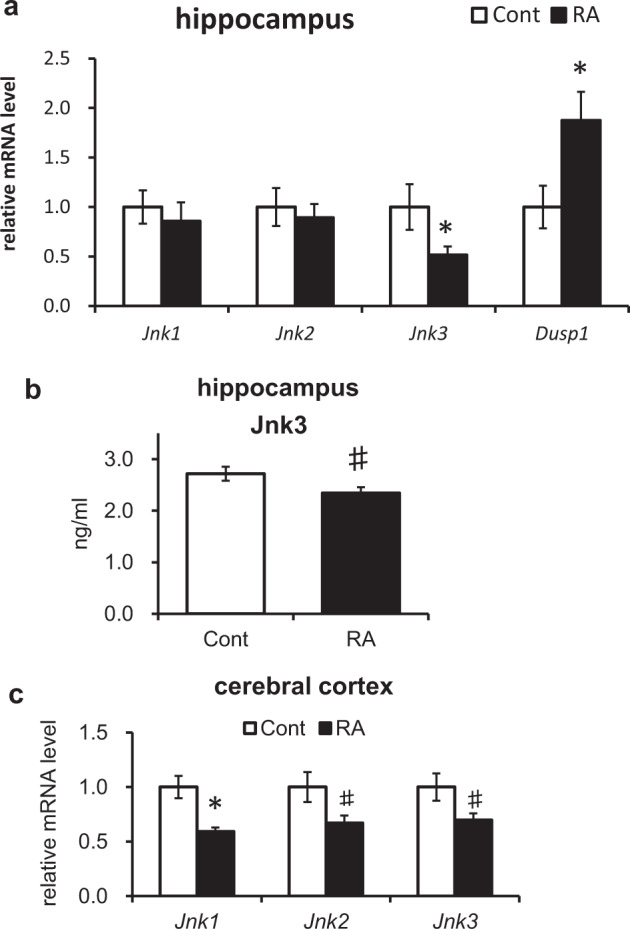
The effects of RA on JNK expression. **a** Quantification of *Jnk* and *Dusp1* genes in hippocampus using quantitative RT-polymerase chain reaction (PCR). **b** Quantification of JNK3 levels in hippocampus using enzyme-linked immunosorbent assay (*p* = 0.077). **c** Quantification of *Jnk* genes in cerebral cortex using qRT-PCR. *Jnk1*, *p* = 0.034; *Jnk2*, *p* = 0.072; *Jnk3*, *p* = 0.066. All mRNA species were quantified relative to the expression of glyceraldehyde-3-phosphate dehydrogenase (*Gapdh*) and presented as fold changes compared to control (Cont). Data are shown as means ± standard error. Cont, *n* = 8; RA, *n* = 9, *p < 0.05, ^#^*p* < 0.10.

Our microarray analysis revealed that, among the tau kinases, *Jnk3* was the most markedly downregulated kinase at mRNA level. Therefore, we analyzed the expression levels of *Jnk* family members (*Jnk1*, *Jnk2*, and *Jnk3*) in the hippocampus and cerebral cortex by qRT-PCR. In agreement with the DNA microarray analysis of the hippocampus, only *Jnk3* expression decreased (Fig. 5a). *Dusp1*, which encodes a highly specific JNK phosphatase, was also upregulated (Fig. 5a). The changes in Jnk3 protein levels were also confirmed by an enzyme-linked immunosorbent assay (Fig. 5b). In the cerebral cortex, all isoforms including *Jnk1* were downregulated (Fig. 5c).

### Feeding with RA inhibits the JNK signaling pathway in the hippocampus

**Fig. 6:**
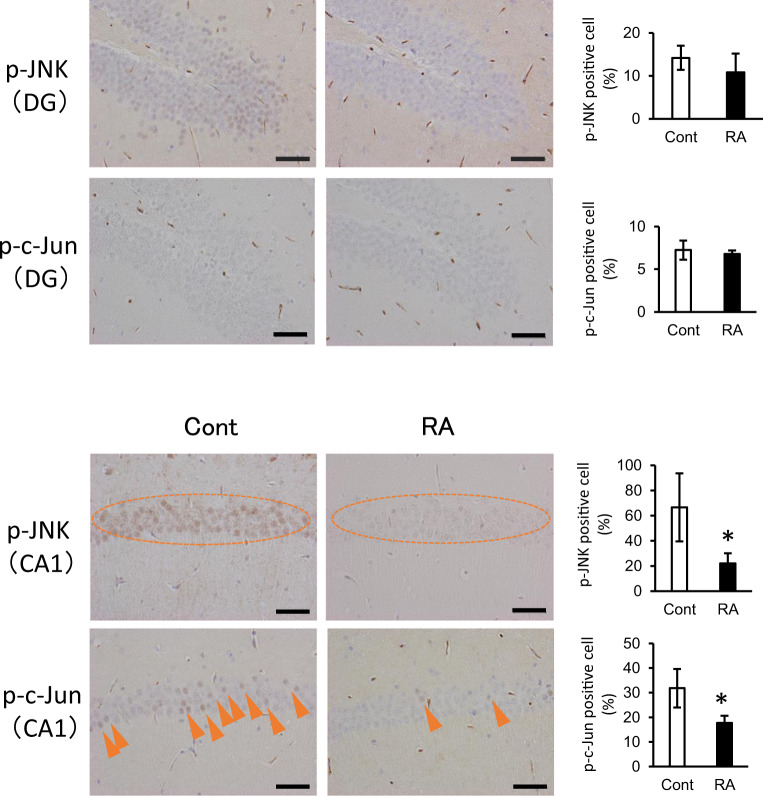
RA inactivates JNK signaling in the hippocampus. Immunohistochemistry for p-JNK and p-c-Jun in the CA1 region. Active JNK (p-JNK) and p-c-Jun expression levels are decreased by RA supplementation. Paraffin sections were stained with anti-p-JNK/p-c-Jun and peroxidase-based labeling (brown), and counterstained with hematoxylin to visualize nuclei (violet). Bregma, −1.8 to −2.2 mm; slice thickness, 5 μm. The CA1 region and dentate gyrus were divided into three areas, and AT8-positive cells were counted to calculate the ratio of cells positive for p-JNK or p-c-Jun to the total number of cells; three mice per group were included in the analyses. The ratio of AT8-positive cells is presented as an average of three areas. Data are shown as means ± standard error. Cont, *n* = 3; RA, *n* = 3, *p < 0.05.

According to immunohistochemical analysis, the expression levels of p-JNK and p-c-Jun decreased in the CA1 region of the RA group relative to expression in the control group (Fig. 6); thus, JNK signaling was apparently attenuated in this area. c-Jun is a representative substrate of JNK, and p-c-Jun is resistant to degradation via proteasomes, leading to its accumulation within NFTs and, thus, its contribution to the tangle maturation process^17^.

### Feeding with RA reduces systemic inflammatory mediator levels

**Fig. 7:**
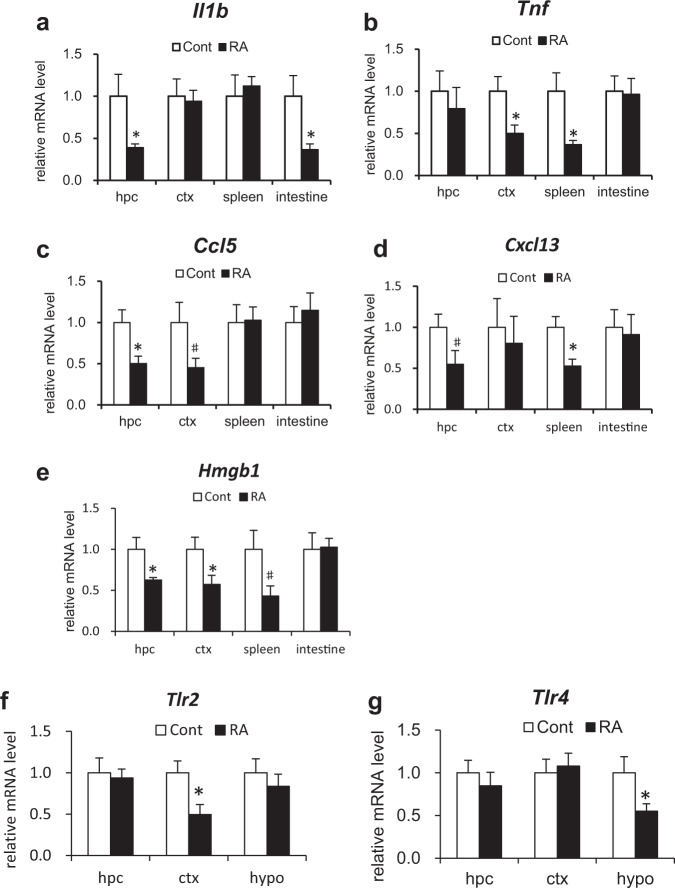
RA reduces systemic inflammation. **a**–**e** Expression levels of inflammatory mediators in hippocampus (hpc), cerebral cortex (ctx), spleen, and small intestine determined by qRT-PCR. *Il-1b* (**a**), *Tnf* (**b**), *Ccl5* (**c**), *Cxcl13* (**d**), and *Hmgb1* (**e**). **f**–**g** Expression levels of *Tlr2/4* in hpc, ctx, and hypothalamus (hypo) determined using qRT-PCR. All mRNA species were quantified relative to the expression of *Gapdh* and presented as fold changes compared to control (Cont). Data are shown as means ± standard error. Cont, *n* = 8; RA, *n* = 9, *p < 0.05, ^#^*p* < 0.10.

We next investigated gene expression levels of the central and peripheral inflammatory mediators as potential factors that alter the activity of stress-associated JNK (Fig. 7a–e). We analyzed hippocampus and cerebral cortex as central sources and spleen and intestines as peripheral sources. In addition to the representative inflammatory mediators, such as interleukin-1β (*Il-1β*) and *Tnf-α*, reductions were observed in the levels of chemokines and damage-associated molecular patterns in the RA group at both the central and peripheral sites (Fig. 7a–e). CCL5 and CXCL13 are strong leukocyte activators, a feature potentially relevant to a range of inflammatory disorders^18^. In the RA group, *Ccl5* and *Cxcl13* expression levels decreased in the hippocampus, cerebral cortex, and spleen (Fig. 7c, d). High mobility group 1 (HMGB1) is a late inflammatory mediator produced in the presence of damaged cells. Compared with the control group, *Hmgb1* expression levels decreased in the hippocampus, cerebral cortex, and spleen of the RA group (Fig. 7e).

Toll-like receptors (TLRs) are activated by exogenous or endogenous ligands to evoke inflammation^19^. Various TLRs are also expressed in neurons, microglia, and astrocytes and contribute to the immunological protection of the central nervous system. Specifically, TLR2 and TLR4 have important roles in sensory and motor functional outcomes during response to neuroinflammation^20^. The expression levels of *Tlr2* in the cerebral cortex and *Tlr4* in the hypothalamus were significantly reduced in the RA group compared with the control group (Fig. 7f, g).

## Discussion

In the present study, we found that RA intake inhibited the pathological hallmarks of AD, including Aβ and p-tau accumulation, and reduced cognitive decline in 3 × Tg-AD mice. The RA group exhibited improved behavioral performance related to spatial working and object memory (Fig. 1). In a review of 3 × Tg-AD mice^21^, the beginning of cognitive index decline in the Y-maze test and novel object recognition test were reported to be 6 and 9 months old, respectively. The Y-maze test was conducted in 10-month-old 3 × Tg-AD mice in this study. The RA group did not affect locomotor activity but improved behavioral performance in the Y-maze test. In Y-maze tests, it has previously been reported that wild-type alteration is ~70%^22^. In the present study, 3 × Tg Cont group alteration was 54 ± 5.14%, which suggests that the spatial memory of the 3 × Tg Cont group was deteriorating. In contrast, 3 × Tg RA group alteration was 67.4 ± 2.44% (Fig. 1d), which is close to that of the wild-type. The novel object recognition test was conducted following an interval of 5 min and 24 h to evaluate short- and long- term memory deficit, respectively^23^. The RA group showed a higher DI when compared with the control group after both 5 min and 24 h intervals (Fig. 1f); thus, both short- and long-term memory functions were apparently improved. From novel object recognition testing, Midel et al.^24^ reported that the DI of wild-type mice was ~0.4 while that of Tg mice was ≤0.2. In the present study, the DIs of the 3 × Tg Cont group (0.1 ± 0.13) and 3 × Tg RA group (0.46 ± 0.35) were similar to those reported by Midel et al. for Tg and wild-type mice, respectively.

After confirming the effect of RA intake on memory improvement in AD model mice based on behavioral tests, we evaluated changes in the pathological hallmarks of AD, including Aβ and p-tau accumulation, by immunohistochemical analysis of the brain. The 3 × Tg-AD mice exhibit Aβ plaques and neurofibrillary tangles after six months of age^14,23^. In the control group, Aβ plaques and p-tau accumulation were found to be mild in the cortical regions in the vicinity of the amygdala and hippocampus, respectively. These data indicate that the control group mice were in an early stage of AD because the AD-associated pathological changes appear earlier in the cortical regions in the vicinity of the amygdala and hippocampus^25^. On the other hand, Aβ plaques and p-tau accumulation in these regions were suppressed in the RA group (Figs 2 and 3), suggesting that RA intake suppressed not only cognitive function but also pathological progression. We have already reported the inhibitory effect of RA on Aβ aggregation in Tg2576 mice, which supports the results of the present study^4^. The decrease in p-tau accumulation in the hippocampal CA1 region based on immunohistochemical staining suggested p-tau particularly strongly involved in suppressing cognitive decline. The hippocampal CA1 region initially recursively outputs to the entorhinal cortex and is crucial in the formation and maintenance of memory. Recent studies have shown that this area is essential for autobiographical memory, autonomous consciousness, and fixation of long-term memory^26,27^. During the pathological progression of AD, CA1 pyramidal cells show p-tau accumulation and neuronal damage earlier than other sites^25^. Furthermore, there is a strong correlation between the extent of p-tau accumulation in hippocampus and the cognitive function score^11^. These results suggest that inhibition of tau phosphorylation in hippocampus is an important mechanism underlying the preventive effect of RA in AD.

The present study revealed that the JNK signaling pathway in inflammation was inhibited in the hippocampus of the RA group (Table 2; Figs 4b, 5, and 6). Phosphorylated JNK was decreased in cells with decreased p-tau expression in the hippocampal CA1 of mice fed an RA-supplemented diet (Figs 3 and 6). RA inhibits JNK activity and protects hippocampal neurons in a mouse model of ischemia/reperfusion injury^28^. Recent studies have indicated that the activation of JNK is associated with the AD pathogenesis; thus, JNK has been garnering attention as a new therapeutic target^29,30^. JNK is activated by various cellular stress stimuli such as inflammatory cytokines, oxidation, and Aβ toxicity^31^. The JNK signaling pathway is a cell death pathway that controls apoptosis signaling^32^. Tau is a substrate of JNK, and the JNK pathway amplifies and drives subcellular changes in tau phosphorylation^33^. JNK upregulation has an complete overlap with p-tau accumulation^34^, as shown in the present study. Amyloid precursor protein is an excellent substrate for JNK, which promotes the production of the highly toxic Aβ^35^. Aβ and p-tau interact with each other; however, their relationship with each other and their common target mechanism of AD prevention is unclear. These data suggested that inhibition of the JNK signaling pathway by RA intake may be an important event for the subsequent suppression of Aβ aggregation and tau phosphorylation.

JNK1, JNK2, and JNK3 have different roles in the brain and phenotypes related to learning and cognition in corresponding knockout mouse models are also distinct^36^. We found that especially of JNK3 was suppressed in the hippocampus with an RA-supplemented diet (Fig. 5). JNK3 is the predominant JNK isoform in the nervous system, and it is highly expressed and activated in the brain tissue and cerebrospinal fluid of patients with AD and significantly correlates with the rate of cognitive decline^37^. Deletion of *Jnk3* in a mouse model of AD was shown to suppress AD-associated symptoms^38^. JNK3 expression is primarily localized to pyramidal neurons in the CA1 region of hippocampus^39^, an area that is vulnerable in early AD^40^.

Furthermore, we found that inflammation was decreased both in the brain and peripheral tissues by RA feeding in the 3 × Tg-AD mice. Recent studies have suggested that neuroinflammation significantly contributes to AD pathogenesis^41^. Most previous anti-inflammatory approaches for AD have been aimed at only reducing central inflammation. A variety of preclinical and clinical studies have demonstrated that peripheral inflammation is communicated to the brain^42–44^. Peripheral inflammatory mediators are upregulated faster than those in the central nervous system and exacerbate the development of AD symptoms^45^. Systemic inflammation in the preclinical stage is closely related to the risk of future cognitive decline^45^. Epidemiological and in vivo studies indicate that anti-inflammatory agents such as nonsteroidal anti-inflammatory drugs could delay the onset and slow the progression of AD^46^. TLRs and cytokine receptors expressed in cerebrovascular endothelial cells mediate the transmission of inflammatory signals to the central nervous system^43^. The suppression of peripheral inflammatory signals, which are considered beneficial for pro-inflammatory signaling in the central nervous system, might therefore potentially contribute to the prevention of AD. RA has potent anti-inflammatory activity in rat models of local and systemic inflammation^47^.

RA feeding suppressed the expression levels of inflammatory mediators, such as *Il-1b*, *Tnf*, *Cxcl13*, and *Hmgb1*, not only in the periphery but also in the brain (Fig. 7a–e). IL-1β and TNF-α, which are typical inflammatory cytokines, are increased in both the brain and periphery and worsen the clinical presentation of early-stage AD^42,48^. The chemokines CCL5 and CXCL13 easily cross the blood–brain barrier, which is compromised by inflammation. HMGB1, a damage-associated molecular pattern and a late inflammatory marker produced in the presence of damaged cells^49^, reduces the ability of microglia to phagocytose Aβ and contributes to the failure of blood–brain barrier function^50,51^. Similar to Aβ, HMGB1 binds to TLR and activates downstream pathways including the JNK signaling pathway^49,52^. Cognitive decline was shown to be suppressed by early subcutaneous injection of HMGB1 antibodies in the 5 × FAD mouse model of AD^53^. In the present study, we confirmed that the inflammatory mediator gene expression levels were decreased in both the periphery and the central nervous system in the RA group, indicating that feeding with RA reduced the chronic inflammatory state, resulting in the suppression of inflammatory signal transmission. Since inflammation activates the JNK signaling pathway, these results suggest that the JNK signaling pathway in hippocampus might be suppressed by the anti-inflammatory effect of RA.

Although we confirmed that inflammation was suppressed in both the periphery and brain, the order in which this suppression took place is unclear. As above discussed, peripheral inflammatory mediators are upregulated faster than those in the central nervous system and exacerbate the development of AD symptoms^45^. Several previous studies have reported that RA has high anti-inflammatory activity in the periphery^54,55^. We previously measured the concentration of RA in the brains and plasma of wild-type mice fed a diet containing 0.5% RA for seven weeks using high-performance liquid chromatography coupled with electrochemical detection^5^. RA was detected in the plasma but not in the brain. Therefore, it is possible that peripheral inflammation may be initially suppressed followed by suppression in the brain. On the other hand, the possibility is that very low concentrations of RA or its metabolites work as an active ingredient in brain. We acknowledge that confirming the order of suppression of inflammation in the periphery and brain and clarifying the pharmacokinetics of RA and its metabolites are important for elucidating the action mechanism of RA; therefore, we will test our hypothesis in future research.

In conclusion, RA supplementation inhibited the pathological hallmarks of AD, including Aβ and p-tau accumulation, and improved cognitive function in male 3 × Tg-AD mice. RA intake also suppressed inflammation in the periphery as well as the central nervous system. As a result, the JNK signaling pathway associated with tau phosphorylation was downregulated, especially in hippocampus. Overall, these data suggest that RA administration suppresses hippocampal JNK signaling pathway and prevents the progression of pathology and cognitive deficits.

## Methods

### Animals

Six to eight-week-old male 3 × Tg-AD mice (The Jackson Laboratory, ME, USA), which express amyloid precursor protein _Swe_, tau_P301L_, and presenilin 1_M146V_, were divided into two groups: control group (*n* = 8) on a normal AIN-93G diet prepared in our laboratory from essential ingredients (Oriental Yeast, Shiga, Japan) and RA group (*n* = 9) on a normal diet supplemented with 0.5% RA. Mice were housed under controlled conditions with a temperature of 20–26 °C and a humidity of 40–60% under a 12/12-h light-dark cycle, with ad libitum food and water intake. Body weight and food consumption were measured twice weekly. After 8 months, all mice were sacrificed under isoflurane anesthesia and blood and organs were collected. The study was approved by the animal experiment committee of the University of Tokyo and performed in accordance with relevant guidelines and regulations (approval number, P16-224).

### Blood biochemistry

Blood samples were centrifuged at 1000 × *g* at 4 °C for 10 min to obtain EDTA plasma. Aspartate aminotransferase, alanine aminotransferase, lactate dehydrogenase, creatine kinase, triacylglycerol, free cholesterol, esterified cholesterol, low-density and high-density cholesterol, glucose, glycoalbumin, and total ketone body levels in plasma were analyzed by Oriental Yeast (Tokyo, Japan).

### Y-maze test

All behavioral tests were performed when the animals were ten months of age. Spontaneous alternation was measured in an acrylic Y-maze comprising three arms (5 cm wide × 30 cm deep × 15 cm high) oriented at an angle of 120° from each other (Fig. 1b). Mice were placed in the distal end of an arm and allowed to explore the maze during an 8-min trial. The sequence of arm entries was recorded and the percentage of alternations (entry into an arm that differed from the previous two entries) was calculated using the following formula: number of alternations/(total number of arm entries − 2).

### Novel object recognition test

The distinction between familiar and unfamiliar objects is an index of recognition memory, and its measurement is aided by the innate preference of rodents for novel objects over familiar objects. The novel object recognition test was performed in a large plastic cage (18 cm wide × 28 cm deep × 22 cm high) and comprised two phases: acquisition phase (phase 1) and test phase (phase 2) (Fig. 1e). In both phases, all mice were placed in the activity chamber and allowed to explore freely for 5 min. In phase 1, the chamber contained two identical objects placed in opposite corners. In phase 2, one of the familiar objects was replaced by a novel object. The objects were validated prior to the test to ensure that there was no inherent preference for either object (data not shown). The two phases were separated from each other by a 5-min and a 24-h interval to evaluate short- and long-term memory, respectively^23^. Exploration was defined as directing the nose to the object at a distance of <2 cm or touching the object with the nose or forepaws. Test results were expressed as discrimination index (DI) between objects during the test session, which was calculated as the difference between the time spent exploring the novel object (N) and the familiar object (F) divided by the total time exploring both objects (DI = [N − F]/[N + F]).

### Immunohistochemistry

Brain hemispheres were embedded in paraffin and cut into 5-μm thick sections. After deparaffinization, the sections were incubated in formic acid for 5 min and in HistoVT One (Nacalai Tesque, Kyoto, Japan) for 20–40 min at 90 °C. After blocking with EzBlock BSA (ATTO, Tokyo, Japan), the sections were incubated with specific primary antibodies overnight at 4 °C. The nucleus of the nerve cells was stained using hematoxylin and eosin staining. The antibodies included mouse monoclonal anti-Aβ 17-24 (4G8, 1:1000; RRID: AB_2564633; BioLegend, CA, USA), mouse monoclonal PHF-tau pSer202/Thr205 (AT8, 1:1000; RRID: AB_223647; Thermo Scientific, IL, USA), mouse monoclonal anti-p-JNK (G-7) pThr183/Tyr185 (G-7, 1:50; RRID: AB_628232; Santa Cruz, TX, USA), and mouse monoclonal anti-p-c-Jun p Ser63 (KM-1, 1:300; RRID: AB_627262; Santa Cruz). The samples were washed with Tris-buffered saline supplemented with 0.03% Triton X-100 and stained using Dako EnVision+ System-HRP labeled polymer (Dako, CA, USA), according to the manufacturer’s instructions. Liquid DAB + Substrate Chromogen system (Dako) was used for visualization according to the manufacturer’s instructions, followed by nuclear counterstaining with Mayer’s hematoxylin. Images were acquired using a light microscope (BX53; Olympus, Tokyo, Japan). Figure 2b shows the Aβ plaque area per 1000 μm^2^ of cortical regions in the vicinity of the amygdala; three mice per group were included in the analyses. As shown in Fig. 3c, d, the CA1 region and dentate gyrus (DG) were divided into three areas, and AT8-positive cells were counted to calculate the ratio of p-tau-positive cells to the total number of cells; three mice per group were included in the analyses. The ratio of AT8-positive cells was presented as an average of three areas.

### DNA microarray analysis

Total RNA was isolated from hippocampus using the NucleoSpin RNA kit (Macherey-Nagel, PA, USA), and RNA quality was determined by RNA integrity number using an Agilent 2100 Bioanalyzer (Agilent Technologies, CA, USA) and an Agilent RNA 6000 Nano kit (Agilent Technologies). The RNA integrity number was >7 for all samples. Affymetrix GeneChip Mouse Genome 430 2.0 arrays (CA, USA) were used for transcriptome expression profiling of hippocampi from four mice with an average weight in each group. The arrays were processed according to the standard protocol included in the GeneChip expression analysis technical manual by Affymetrix. Briefly, the pooled total RNA was reverse transcribed into double-stranded cDNA using the Affymetrix GeneChip 3′ IVT Express kit (Thermo Fisher Scientific), and labeled DNA aliquots were incubated in the hybridization solution at 45 °C for 16 h. After washing and staining, the arrays were scanned using the Affymetrix GeneChip Scanner 3000. The microarray dataset has been submitted to the Gene Expression Omnibus (https://www.ncbi.nlm.nih.gov/geo/) under the accession number GSE128494. The CEL files were analyzed using the statistical software program R (https://cran.r-project.org/) and Bioconductor 2.2 (http://www.bioconductor.org/). Signal intensity was normalized using the FARMS method^56^ with R 2.10.1, and probe sets were extracted based on a false discovery rate of 0.05 for differentially expressed genes (DEGs) by the rank product method^57^ using R 3.1.2. Between-sample similarity was examined using principal component analysis. DEGs were identified with gene ontology (GO) terms using the Database for Annotation Visualization and Integrated Discover (DAVID) 6.8 (https://david.ncifcrf.gov) based on a *p* value of < 0.005 by the modified Fisher’s exact test and a fold enrichment score of >3.0.

### Quantitative RT-PCR

Quantitative reverse transcription (qRT)-polymerase chain reaction (PCR) was performed for samples collected from all animals in both groups (control, *n* = 8; RA, *n* = 9). Briefly, purified total RNA (0.5 μg) was reverse transcribed using the PrimeScript RT Reagent kit (TaKaRa, Shiga, Japan) and the synthesized cDNA was amplified on a Rotor-Gene 6000 instrument (Corbett Research, Sydney, Australia) using TB Green Premix EX Taq II (TaKaRa). Primers (Supplementary Table S1) were designed using the PRIMER3 web application. SYBR green EX (TaKaRa) was used on a real-time PCR detection system (TaKaRa). Relative mRNA amounts were normalized to glyceraldehyde-3-phosphate dehydrogenase (*Gapdh*) mRNA and expressed as fold changes.

### Enzyme-linked immunosorbent assay

Neuronal protein extract reagent (Thermo Scientific) was added to frozen hippocampal tissues and homogenized using BioMasher II (Nippi, Tokyo, Japan). The homogenates were centrifuged at 10,000 × *g* for 4 °C at 10 min to obtain supernatants. c-Jun N-terminal kinase 3 (JNK3) levels were quantified using the mouse mitogen-activated protein kinase 10 enzyme-linked immunosorbent assay kit (MyBioSource, CA, USA) according to the manufacturer’s instructions. Absorbance at 450 nm was measured using a microplate reader (Varioskan LUX; Thermo Fisher Scientific), and JNK3 concentrations (ng/mL) in samples were calculated (control, *n* = 5; RA, *n* = 6).

### Statistical analysis

Values were expressed as means ± standard error. Data between two groups were compared using Student’s *t-*test, and the differences were considered statistically significant at a *p-*value of < 0.05.

## Supplementary information

Supplemental materials

reporting-summary

## Data Availability

The data supporting the findings reported herein are available on reasonable request from the corresponding author.
