# Supplementary material for: Rosmarinic acid suppresses tau phosphorylation and cognitive decline by downregulating the JNK signaling pathway
Source: NPJ Sci Food. 2021 Jan 29;5:1. doi: 10.1038/s41538-021-00084-5 (PMC7846760; doi:10.1038/s41538-021-00084-5)
Supplement: Supplementary file 1 — Supplemental materials [file 41538_2021_84_MOESM1_ESM.pdf]

Supplemental materials

Rosmarinic acid suppresses tau phosphorylation and cognitive decline by downregulating the JNK signaling pathway

So Yamamoto[a], Tomoko Kayama[a], Moeko Noguchi-Shinohara[b], Tsuyoshi Hamaguchi[b], Masahito Yamada[b], Keiko Abe[c,d], Shoko Kobayashi[a]*

[a]Research Center for Food Safety, Graduate School of Agricultural and Life Sciences, The University of Tokyo, 1-1-1 Yayoi, Bunkyo-ku, Tokyo 113-8657, Japan

[b]Department of Neurology and Neurobiology of Aging, Kanazawa University Graduate School of Medical Sciences, Kanazawa 920-0934, Japan

[c]Department of Applied Biological Chemistry, Graduate School of Agricultural and Life Sciences, The University of Tokyo, Yayoi, Bunkyo-ku, Tokyo 113-8657, Japan

[d]Group of Food Functionality Assessment, Kanagawa Institute of Industrial Science and Technology, Life Science Environment Research Center, Tonomachi, Kawasaki, Kanagawa, 210-0821, Japan

Supplementary Table S1. All primer sequences used in this study

Gene	Sequence (Forward)	Sequence (Reverse)
Gapdh	5'-GTCGGTGTGAACGGATTTGG-3'	5'-GACTCCACGACATACTCAGC-3'
Jnk1	5'-TGTTCCCCGATGTGCTTTTC-3'	5'-ACGTTGATGTATGGGTGCTG-3'
Jnk2	5'-GGCATGGGCTACAAAGAGAATG-3'	5'-AACTCTGCGGATGGTGTTCC-3'
Jnk3	5'-GTCCTCATGAAGTGTGTGAACC-3'	5'-AGCTCCATCTGAATCACCTGAC-3'
Dusp1	5'-TACTAGTGTGCCTGACAGTGC-3'	5'-AGCATATCCTTCCGAGAAGCG-3'
Il1b	5'-AAGTTGACGGACCCCAAAAG-3'	5'-ATGTGCTGCTGCGAGATTTG-3'
Tnf	5'-AGCCTCTTCTCATTCCTGCTTG-3'	5'-TGGGCCATAGAACTGATGAGAG-3'
Ccl5	5'-TCACCATCATCCTCACTGCAG-3'	5'-AGAGGTAGGCAAAGCAGCAG-3'
Cxcl13	5'-GCTGCCCCAAAACTGAAGTTG-3'	5'-TTTGGCACGAGGATTCACAC-3'
Hmgb1	5'-ACAACACTGCAGCAGATGAC-3'	5'-TTCGCTGCATCAGGTTTTCC-3'
Tlr2	5'-CGTTGTTCCCTGTGTTGCT-3'	5'-AAAGTGGTTGTCGCCTGCT-3'
Tlr4	5'-TTCACCTCTGCCTTCACTACA-3'	5'-GGGACTTCTCAACCTTCTCAA-3'

Supplementary Table S2. Plasma biochemistry

test	Control	RA	*p*-value
AST (IU/L)	86.0 ± 9.5	100.8 ± 8.3	0.27
ALT (IU/L)	26.0 ± 4.1	30.8 ± 4.0	0.43
LDH (IU/L)	777.2 ± 341	707.4 ± 119	0.83
CK (IU/L)	148.4 ± 23.0	244.8 ± 36.3	0.07
TG (mg/dL)	50.0 ± 7.1	43.7 ± 10.0	0.65
F-CHO (mg/dL)	12.0 ± 0.9	13.4 ± 2.2	0.62
E-CHO (mg/dL)	28.0 ± 4.4	33.1 ± 6.4	0.56
LDL-C (mg/dL)	2.8 ± 0.5	2.8 ± 0.4	0.93
HDL-C (mg/dL)	20.8 ± 2.6	26.2 ± 4.2	0.34
GLU (mg/dL)	286.4 ± 18.6	290.8 ± 15.8	0.86
GA (%)	5.2 ± 0.5	5.8 ± 0.4	0.35
T-KB (μmol/L)	82.4 ± 6.9	100.8 ± 11.1	0.23

Values are expressed as the mean ± S.E. (n = 8) AST: aspartate aminotransferase, ALT: alanine aminotransferase, LDH: lactate dehydrogenase, CK: creatine kinase, TG: triglyceride, F-CHO: free cholesterol, E-CHO: esterified cholesterol, LDL-C: low-density lipoprotein cholesterol, HDL-C: high-density lipoprotein cholesterol, GLU: glucose, GA: glycoalbumin, T-KB: total ketone body.

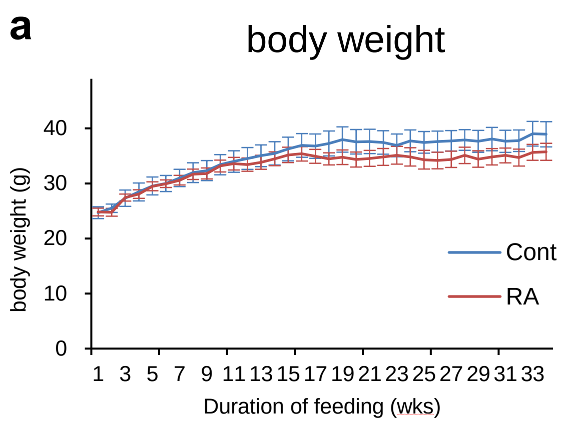

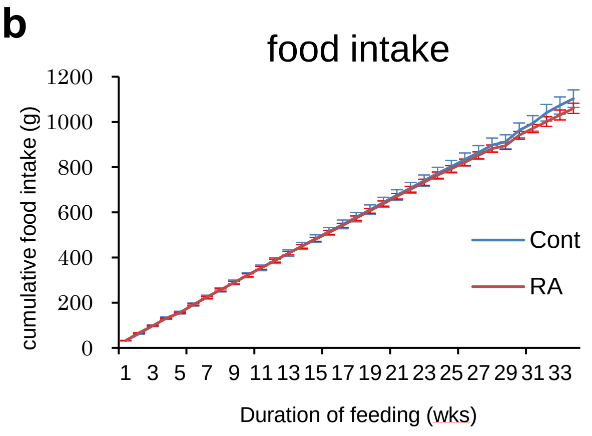

Supplementary Figure S1. Body weight and food intake. Body weight and food consumption were measured twice a week following receipt of animals and for a period of 8 months. ***a*** Body weight. ***b*** Cumulative food intake. Data are shown as mean ± S.E.
